# Structural optical and electrical properties of a transparent conductive ITO/Al–Ag/ITO multilayer contact

**DOI:** 10.3762/bjnano.11.57

**Published:** 2020-04-27

**Authors:** Aliyu Kabiru Isiyaku, Ahmad Hadi Ali, Nafarizal Nayan

**Affiliations:** 1 Optical Fiber Laser Technology Group, Department of Physics and Chemistry, Faculty of Applied Sciences and Technology Pagoh Educational Hub, University Tun Hussein Onn Malaysia, 84600 Pagoh, Johor, Malaysia; 2 Department of Physics, Faculty of Science, Kaduna State University, P.M.B 2339, Kaduna State, Nigeria; 3 Microelectronic and Nanotechnology Shamsuddin Research Centre (MiNT-SRC), Universiti Tun Hussein Onn Malaysia, 86400 Parit Raja, Batu Pahat, Johor, Malaysia

**Keywords:** annealing, DC sputtering, figure of merit, indium tin oxide (ITO), multilayer structure, RF sputtering

## Abstract

Indium tin oxide (ITO) is a widely used material for transparent conductive oxide (TCO) films due to its good optical and electrical properties. Improving the optoelectronic properties of ITO films with reduced thickness is crucial and quite challenging. ITO-based multilayer films with an aluminium–silver (Al–Ag) interlayer (ITO/Al–Ag/ITO) and a pure ITO layer (as reference) were prepared by RF and DC sputtering. The microstructural, optical and electrical properties of the ITO/Al–Ag/ITO (IAAI) films were investigated before and after annealing at 400 °C. X-ray diffraction measurements show that the insertion of the Al–Ag intermediate bilayer led to the crystallization of an Ag interlayer even at the as-deposited stage. Peaks attributed to ITO(222), Ag(111) and Al(200) were observed after annealing, indicating an enhancement in crystallinity of the multilayer films. The annealed IAAI film exhibited a remarkable improvement in optical transmittance (86.1%) with a very low sheet resistance of 2.93 Ω/sq. The carrier concentration increased more than twice when the Al–Ag layer was inserted between the ITO layers. The figure of merit of the IAAI multilayer contact has been found to be high at 76.4 × 10^−3^ Ω^−1^ compared to a pure ITO contact (69.4 × 10^−3^ Ω^−1^). These highly conductive and transparent ITO films with Al–Ag interlayer can be a promising contact for low-resistance optoelectronics devices.

## Introduction

Transparent conducting oxides (TCO) thin films have been receiving much attention regarding their use as contacts in several optoelectronic devices such as LEDs [1], solar cells [2] and flat panel displays [3]. Indium tin oxide (ITO) is the most commonly used TCO for industrial and laboratory applications due to its excellent optical and electrical properties [4–5]. It is a wide-bandgap material (3.6–4.0 eV) with low electrical resistivity. ITO contains the rare and expensive metal indium, which is reflected in the market value of the material [6]. Hence, a reduction of the ITO consumption is desirable. ITO films with smaller thickness would result in high optical transmittance in the visible region. However, the resistivity would increase, which is an issue [4,7–8]. Therefore, the search for new material compositions and structures of ITO-based films to enhance the performance in optoelectronic devices is of importance. The inclusion of a thin metal film between a top and a bottom ITO layer to form a multilayer structure has been explored recently for efficient photoelectric devices [7]. The multilayer structure not only improves the conductivity of the contact but also make the device cost-effective since less indium metal is needed [9–11]. The insertion of a metal layer reduces the transparency of the ITO electrode due to opaqueness of the metal, but selecting an optimal metal thickness can effectively decrease the reflection from the metal film and thus enhance the transmittance. Furthermore, it gives room for controlling the transparency in the visible region of the light spectrum [9,12–13]. However, the quality of both metal and ITO layers determines the optical and electrical performance of the multilayer structures [4].

Embedding a thin metal film between ITO layers coupled with annealing enhances the photoresponse and the rectification properties of the ITO device. Single or double metal thin films of Ag, Al, Ti, Au, Cr, or Ni have been embedded between ITO layers [4,8,14–17]. Free electrons in the metal/ITO materials accelerate the separation of charge carriers and hence improve the transport from the lower to the upper part of the device [9]. The good adhesion, low resistivity, and the stability against oxidation and corrosion of Al films make them suitable for application in optical and electronic devices [18–20]. The low resistivity and relatively high transmittance (compared to other metals) in the visible region of Ag thin films at room temperature led to the wide use of Ag layers in ITO multilayer contacts [21–24]. However, Ag thin films agglomerate upon annealing due to low adhesion, which degrades the quality of the films [25]. This issue can be overcome by adding a thin layer of Al, Au, Pd, or Cr to the Ag film to improve the adhesion [4,25–26].

Optical and electrical properties of ITO films are enhanced by post-deposition annealing especially at high temperatures [7]. Gulen et al. [27] exposed pure ITO films deposited by sputtering to heat treatment at temperatures of 100–700 °C. An improvement of the microstructural, optical and electrical properties of the film annealed at 400 °C was observed. Similarly, a significant enhancement of the optoelectronic properties of an ITO/Ag(Cr)/ITO multilayer film was achieved by annealing at 500 °C [4]. Further treatments beyond 500 °C resulted in the degradation of the film structure due to the appearance of metallic nanoparticles on the surface of the multilayer [4,28]. Furthermore, Cho et al. estimated a figure of merit of 12.28 × 10^−4^ Ω^−1^ for a 5.07 nm thick intermediate Al film after annealing at 200 °C [29]. Rapid thermal annealing of ITO/Ag/ITO films by Joeng et al. [28] led to an improvement in transmittance for films annealed at 300 °C. The lowest sheet resistance and resistivity values were obtained after annealing at 500 °C, but with reduced optical transmittance. Also, a durability test of an ITO sandwich electrode with Ag alloy against heat treatment at 450 °C was carried out by Roh et al. [30]. An appreciable durability and stability of the Ag films was observed. In the present work, the structural, optical and electrical properties of an Al–Ag bilayer between ITO layers (ITO/Al–Ag/ITO) are examined. Moreover, annealing was carried out at 400 °C with an ITO/Al–Ag/ITO (IAAI) multilayer film and a pure ITO film for comparison.

## Results and Discussion

**Figure 1:**
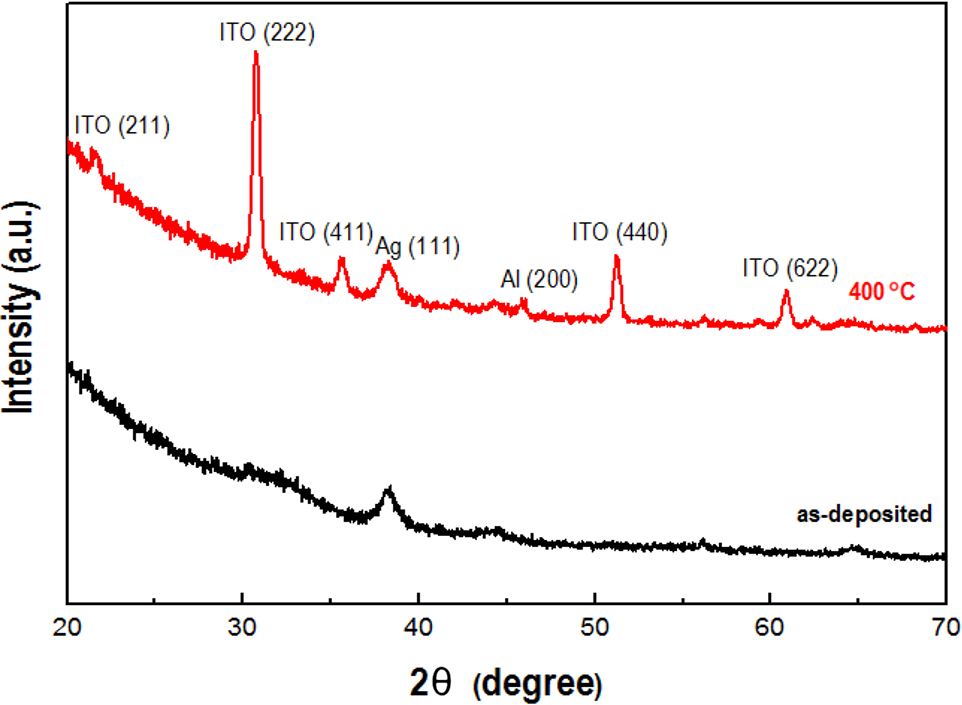
XRD spectra of as-deposited and annealed IAAI films.

Figure 1 shows the X-ray diffraction (XRD) patterns for as-deposited and annealed IAAI multilayer films. The as-deposited film shows an amorphous structure of the top ITO layer with a strong Ag(111) diffraction peak, showing that the Ag intermediate layer is crystalline, comparable to the work of Kim et al. [31]. There is no diffraction peak of the Al film, which is consistent with the work of Cho et al. [29]. The IAAI film becomes polycrystalline upon annealing at 400 °C. Strong diffraction peaks of ITO(222), Ag(111) and ITO(440) were observed after annealing. The appearance of diffraction peaks of ITO(222), Ag(111) and Al(200) in the annealed film indicate an enhanced crystallinity of the film. Diffraction peaks of In_2_O_3_ appear to be dominant without any traces of SnO_2_, Sn or SnO peaks.

During deposition of the IAAI films, the kinetic energy of the sputtered atoms arriving at the substrate is low, which leads to the amorphous structure. The kinetic energy of the Ag atoms is higher and the Ag film crystallizes already during deposition. During annealing the adatoms gain additional energy resulting in an increased mobility. This enables grain growth and crystallization [32–34]. The grain sizes of as-deposited and annealed IAAI films were calculated using the Scherrer equation,

[1] D=0.9λ/βcosθ ,

where λ is the wavelength of the X-ray radiation (here Cu Kα, λ = 1.5406 Å), β is the corrected full width at half-maxima (FWHM) and θ is the Bragg angle. The calculated grain sizes are 51.6 nm for the as-deposited IAAI film and 68.9 nm for the annealed IAAI film.

**Figure 2:**
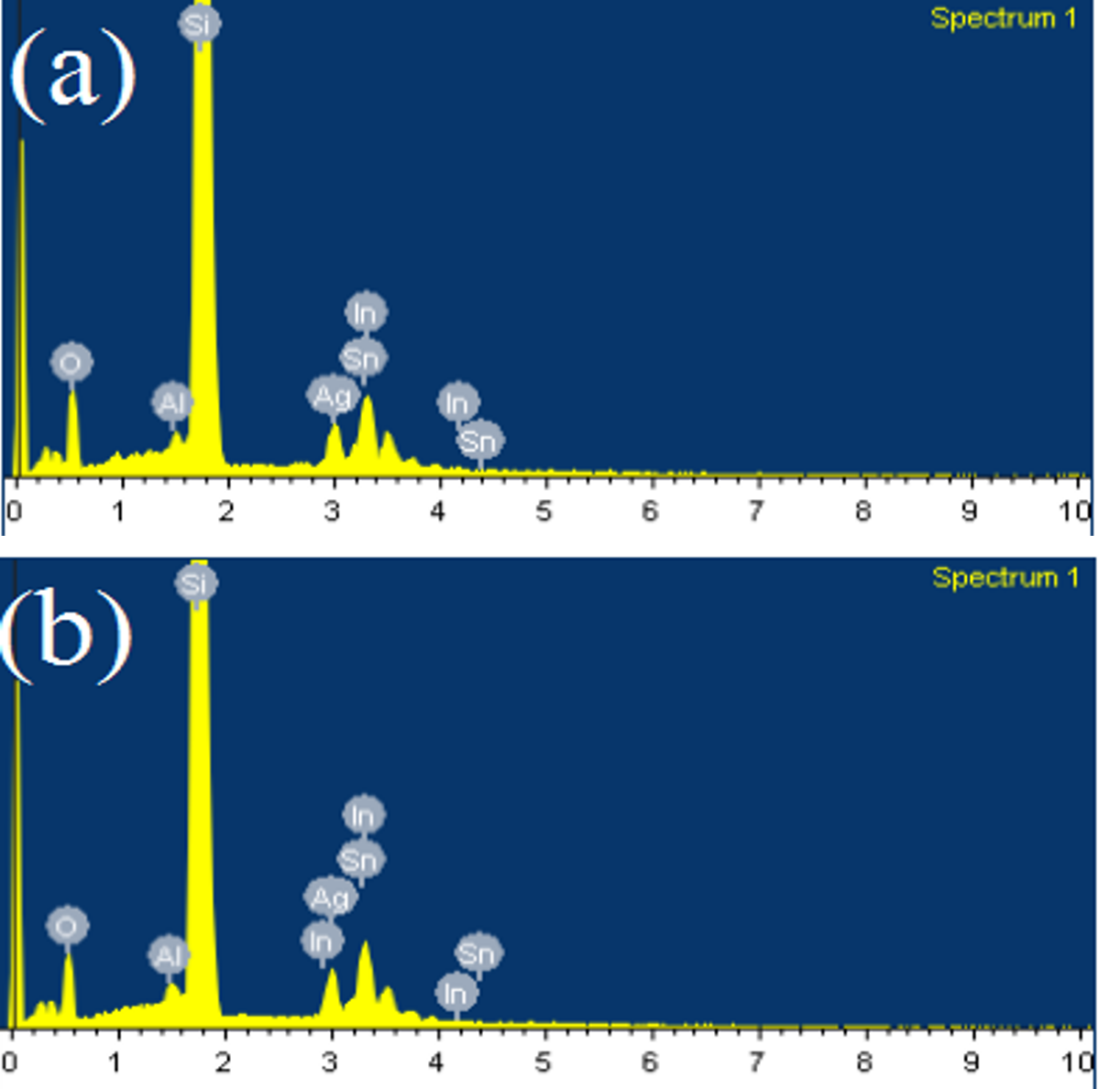
EDXS spectra of (a) the as-deposited IAAI film and (b) the annealed IAAI film.

The elemental composition (wt %) of the IAAI films before and after annealing on the Si substrate obtained using energy-dispersive X-ray spectroscopy (EDXS) are displayed in Table 1. A reduction of the oxygen content was observed after annealing. The content of metal and semiconductor materials accordingly increases. Si as substrate material shows the highest content. The fraction of Sn is low because it is the dopant element in ITO. The low content of Al is attributed to the very thin layer. The EDXS spectra of the films before and after annealing are shown in Figure 2.

**Table 1:** Elemental composition of the IAAI films before and after annealing on the silicon substrate.

	O (wt %)	Al (wt %)	Ag (wt %)	Si (wt %)	In (wt %)	Sn (wt %)

as-deposited IAAI film	12.20	0.35	5.70	69.46	11.04	1.25
annealed IAAI film	10.01	0.55	5.81	71.15	11.17	1.31

**Figure 3:**
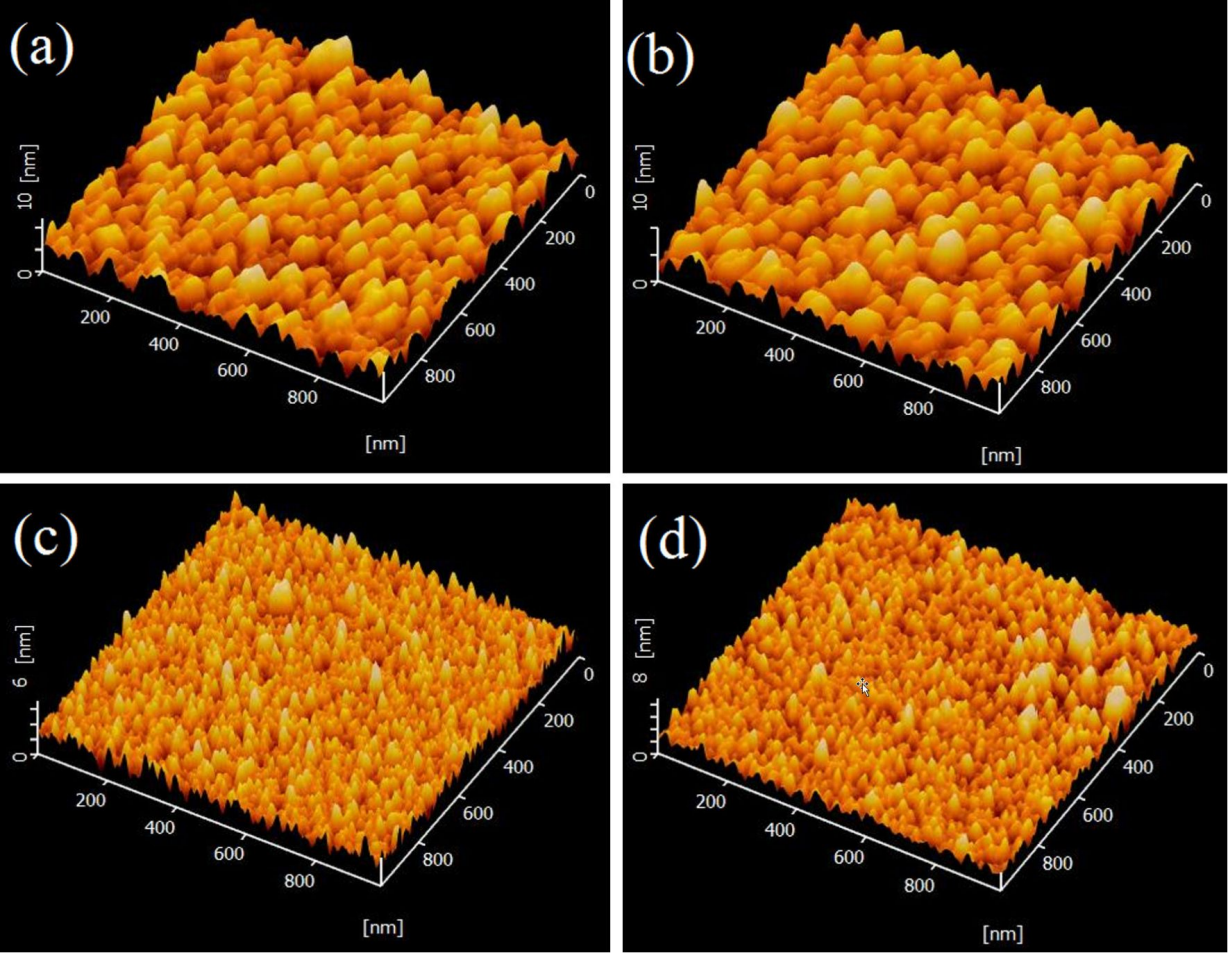
AFM 3D images of (a) the as-deposited IAAI film, (b) the annealed IAAI film, (c) the as-deposited ITO film and (d) the annealed ITO film.

The surface morphology of the IAAI and ITO films was studied using atomic force microscopy (AFM) of an area of 1 μm × 1 μm as shown in Figure 3. A low surface roughness with increased root mean square (*R*_rms_) and average roughness (*R*_a_) values for both IAAI and ITO films after annealing were observed. As determined using the Nanoscope Analysis software, the average grain size increased from 53.53 nm (as-deposited) to 60.03 nm (annealed) for the IAAI film and from 27.59 nm (as-deposited) to 31.18 nm (annealed) for the ITO film. Similarly, after annealing treatment, the IAAI *R*_rms_ and *R*_a_ roughness values increased from 1.569 nm and 1.257 nm to 1.663 nm and 1.339 nm, respectively. The increase in surface roughness is attributed to the increasing grain sizes [35]. The large grain sizes of IAAI films reduce the number of grain boundaries and thus the scattering at grain boundaries. This improves the carrier mobility leading to an increased electrical conductivity of the films.

**Figure 4:**
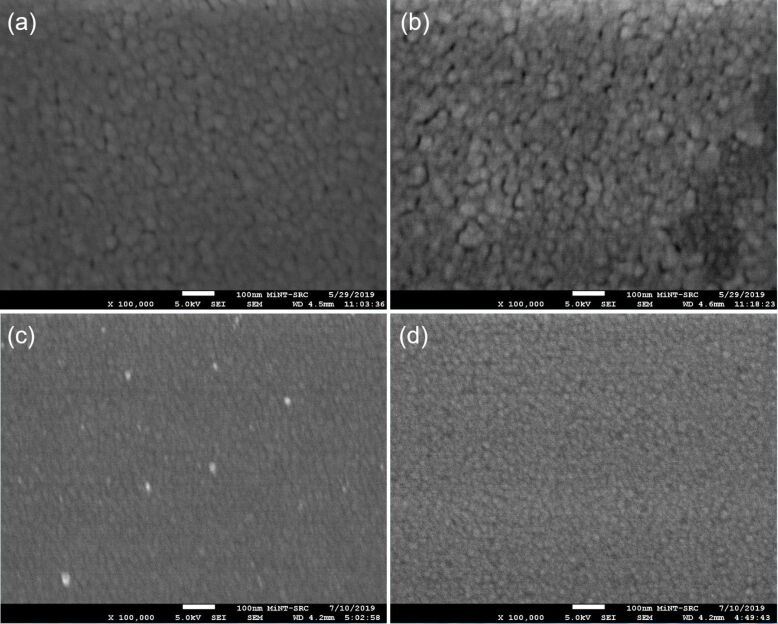
FESEM images of (a) the as-deposited IAAI film, (b) the annealed IAAI film, (c) the as-deposited ITO film and (d) the annealed ITO film.

The surface morphology of the IAAI and ITO films was further analyzed by field-emission scanning electron microscopy (FESEM, 5.0 kV, 100000× magnification). Figure 4 shows FESEM images of the as-deposited and annealed IAAI and ITO films. The as-deposited IAAI and ITO films show a smooth and continuous surface with small grains and dense particles. After annealing, the film surfaces become smoother and the grain sizes increase, with the IAAI films exhibiting larger grain sizes. Island formation or agglomeration of the Al–Ag metal film on the surface of the annealed IAAI film was not observed. Hence, the IAAI structure is stable at this annealing temperature. The films surface roughness results obtained from FESEM and AFM are in good agreement.

**Figure 5:**
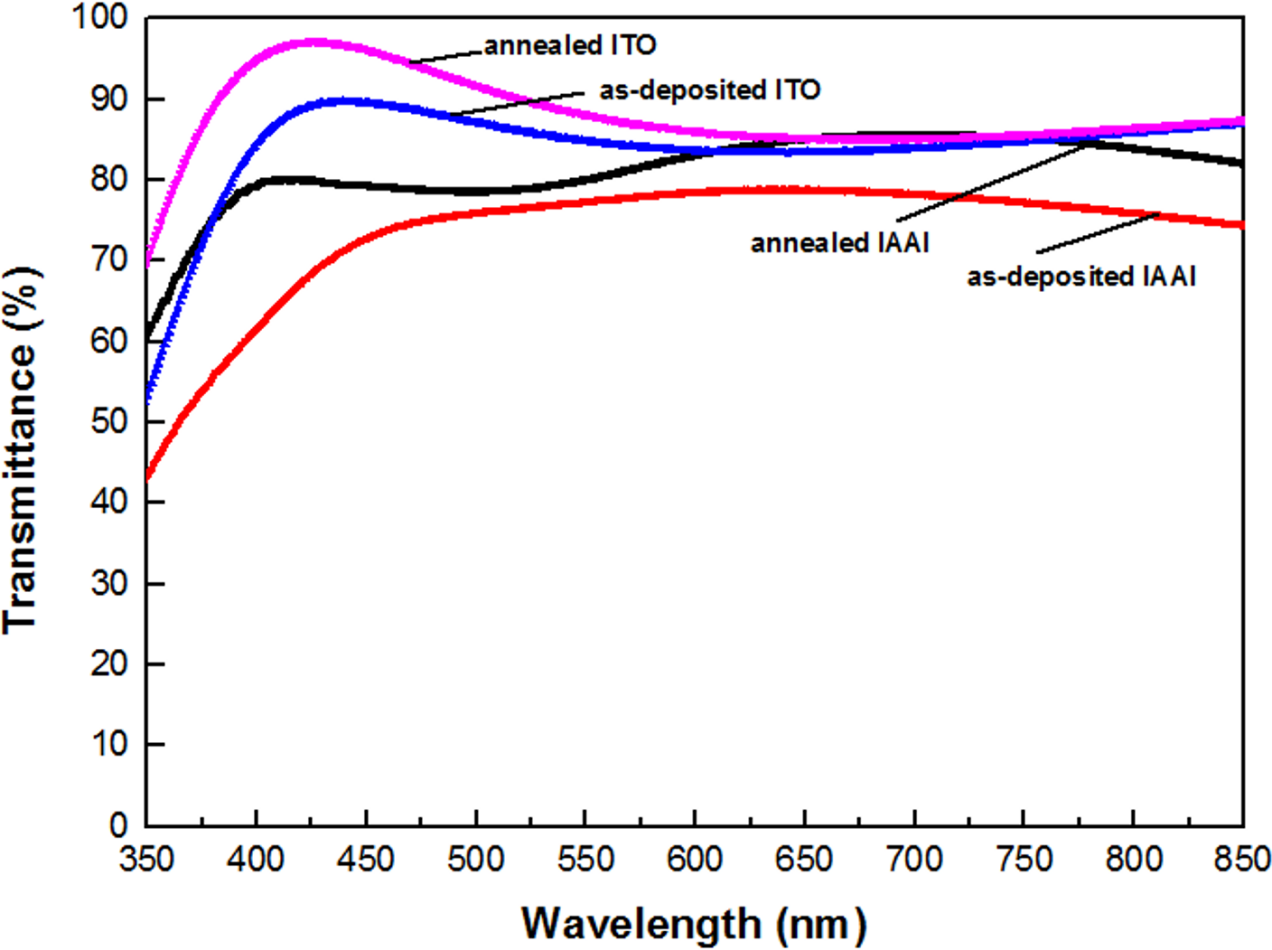
Optical transmittance of as-deposited and annealed IAAI and ITO films.

The optical characteristics of the as-deposited and annealed IAAI and ITO films measured by UV–vis spectrophotometry are shown in Figure 5. It can be seen, that the annealed IAAI and ITO films show a significant increase in optical transmittance. The annealed IAAI film has a transmittance of ca. 86.1% in the visible range. The increase in transmittance is attributed to the improvement of the crystallinity of both the ITO film and the metal interlayer after annealing, which resulted in less light scattering in the metal interlayer [28–30]. The annealing treatment has successfully reduced the number of defects leading light scattering [4,36]. The enhanced structural ordering decreases the electron scattering at grain boundaries and impurities. This can lead to an increase in the effective charge-carrier conduction. Although the annealed ITO film shows a higher transmittance of ca. 96%, this comes with an increasing resistivity [32,37]. It can be observed in Figure 5, that the transmittance spectrum of the annealed IAAI film is shifted toward shorter wavelengths. The increase in carrier concentration in the ITO film is responsible for the blue shift and this can be clearly explained by the Burstein–Moss shift model [9,38].

Electrical properties of IAAI and ITO films obtained from four-point probe and Hall effect measurements are given in Table 2. Sheet resistance and resistivity of IAAI films are lower than those of the annealed ITO film and further decrease upon annealing at 400 °C in air. The decrease can be attributed to the inclusion of the highly conductive metal layers. Even though the inclusion of the metal layers decreases the optical transmittance of the films, the heat treatment lowers the trade-off between transparency and conductivity by reducing structural defects in the films. The sheet resistance of the multilayer structure of the IAAI film can be expressed as [29]:

[2] $$\frac{1}{R_{{\text{s}}^{\text{IAAI}}}}=\left(\frac{1}{R_{\text{Al}}}+\frac{1}{R_{\text{Ag}}}\right)+\frac{2}{R_{{\text{s}}^{\text{ITO}}}}\;,$$

where 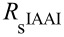 is the total sheet resistance of the IAAI film, *R*_Al_ and *R*_Ag_ are the sheet resistance values of the Al and the Ag film, respectively, and 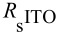 is the assumably equal sheet resistance value of the top and the bottom ITO layer.

The lowest sheet resistance and resistivity values of 2.93 Ω/sq and 2.64 × 10^−5^ Ω·cm, respectively, were obtained from the annealed IAAI film as indicated in Table 2. Generally, the inclusion of low-resistivity metal thin films, coupled with a reduction in grain boundary scattering after annealing, is responsible for the large reduction in both sheet resistance and resistivity. Moreover, the optoelectronic properties of the IAAI films are better compared to those in the works of Roh et al. [30] and Ding et al. [39] who used aluminum/palladium and copper interlayers, respectively. Carrier concentration and mobility of the IAAI films are higher than those of the ITO films. The IAAI carrier concentration increased from 6.2 × 10^−21^ to 8.9 × 10^−21^ cm^−3^ after annealing. Likewise, the carrier mobility increased significantly from ca. 22.5 cm^2^ V^−1^ s^−1^ to ca. 30.2 cm^2^ V^−1^ s^−1^. Similar findings were reported by Meshram et al. [4], Kumar et al. [9] and Ali et al. [34]. The increased carrier concentration is attributed to grain growth and decreased scattering.

**Table 2:** Comparison of electrical resistivity, sheet resistance, carrier concentration, and carrier mobility of as-deposited and annealed ITO and IAAI multilayer films.

	as-deposited ITO	annealed ITO	as-deposited IAAI	annealed IAAI

resistivity (Ω·cm)	1.64 × 10^−4^	1.52 × 10^−4^	8.12 × 10^−5^	2.64 × 10^−5^
sheet resistance (Ω/sq)	18.2	9.34	8.12	2.93
carrier concentration (cm^−3^)	2.01 × 10^21^	2.58 × 10^21^	6.2 × 10^21^	8.9 × 10^21^
mobility (cm^2^·V^−1^·s^−1^ )	2.75	5.51	22.5	30.2

The quality of any ITO-based film is given by a high optical transmittance *T*_opt_ and a low sheet resistance *R*_s_. The quality of IAAI multilayer and ITO films were determined using the figure of merit (FOM, Equation 3) developed by Haacke [40]:

[3] $$\text{FOM}=\frac{T_{\text{opt}}{}^{10}}{R_{\text{s}}}\;.$$

**Figure 6:**
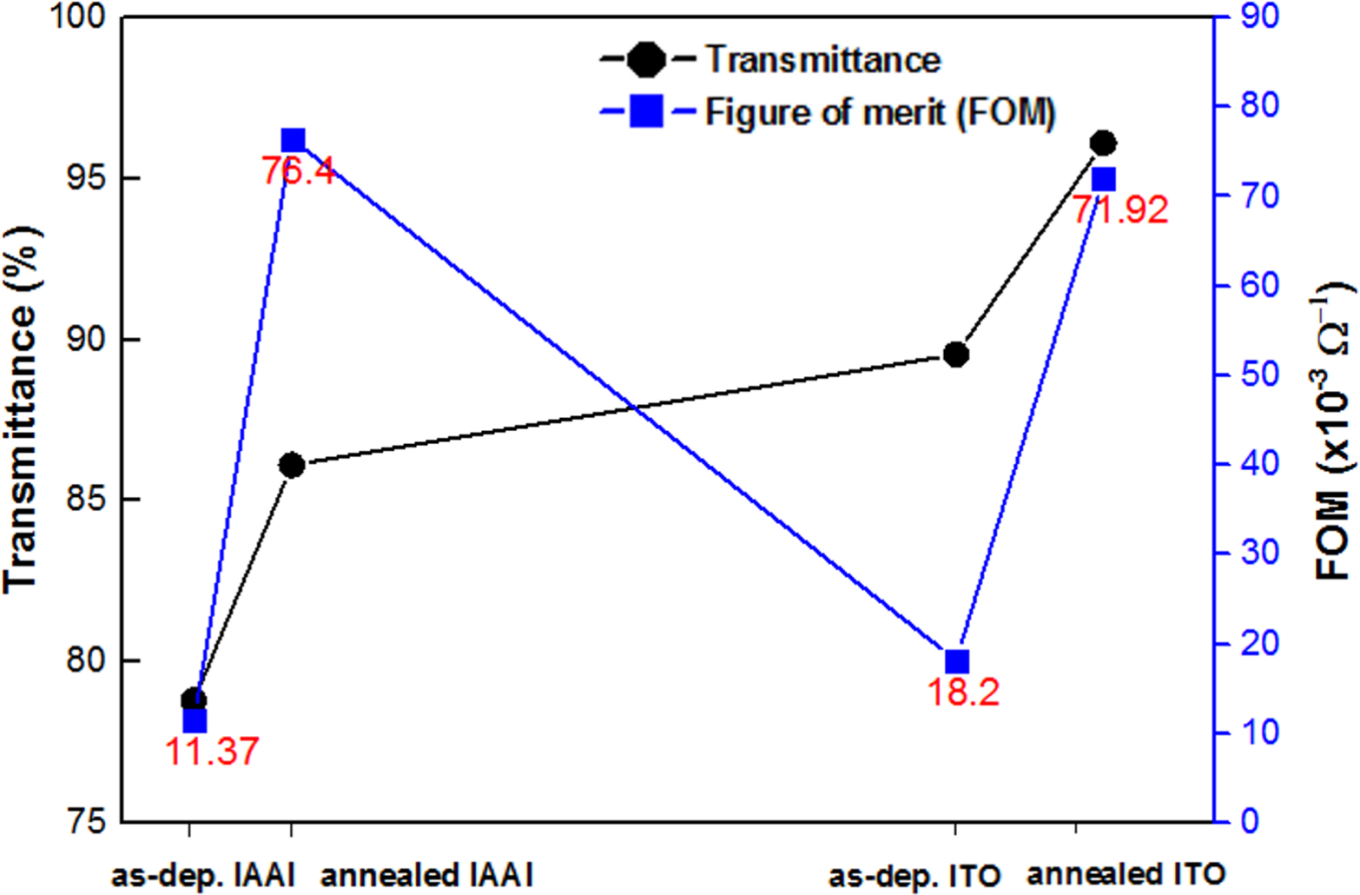
Transmittance and FOM of as-deposited and annealed IAAI multilayer and ITO films.

Figure 6 shows the transmittance and FOM values of as-deposited and annealed IAAI and ITO films. The FOM of the annealed IAAI film rose to 76.4 × 10^−3^ Ω^−1^ compared to 71.92 × 10^−3^ Ω^−1^ for the annealed ITO film. The FOM value of the annealed IAAI film indicates a significant improvement compared those in the works of Kumar et al. [9] and Meshram et al. [4] in which nickel and silver/chromium interlayers were used, respectively. The combination with Ag and Al metal films coupled with annealing at 400 °C have significantly improved the performance of the ITO-based films as determined by FOM [9,30]. This is due to the reduction in sheet resistance accompanied by the enhanced optical transmittance.

## Conclusion

ITO/Al-Ag/ITO (IAAI) multilayer films were deposited by RF and DC magnetron sputtering at room temperature. The inclusion of the Al–Ag bilayer coupled with annealing at 400 °C significantly enhanced the microstructural, optical and electrical properties of the multilayer films. A high optical transmittance of 86.1% at 450 nm with a low sheet resistance of 2.93 Ω/sq was obtained for the annealed IAAI film. The figure of merit of the annealed IAAI film (76.4 × 10^−3^ Ω^−1^) is higher than that of a pure ITO film (71.9 × 10^−3^ Ω^−1^). The improvement of the optoelectronic properties of the IAAI film is attributed to the annealing treatment. The resulting larger grain sizes and lower surface roughness of the IAAI film lead to the enhanced optical and electrical properties.

## Experimental

### Materials

A commercial In_2_O_3_/SnO_3_ (ITO) target of 90:10 weight ratio was used for the deposition of ITO films. Aluminium (Al) and silver (Ag) targets of 99.999% purity were purchased and used for the deposition of the Al–Ag interlayer between top and bottom ITO layers. Silicon wafers [Si(100), n-type, phosphorous, 1–10 Ω·cm and 525 ± 25 μm] or commercial soda-lime glass (for optical measurements) were used as substrates. Decon90 glass cleaner was used for glass substrate cleansing.

### Thin film preparation

A SNTEK Korea magnetron sputtering system with a dual radio frequency (RF)/direct current (DC) sputtering source with a main deposition chamber 15.7 inches in height and 23.6 inches in diameter was used for the thin films preparation. The top and bottom ITO layers were deposited using RF sputtering while DC sputtering was applied for the deposition of the Al–Ag bilayer. Prior to deposition, Si substrates were heated in acetone at 55 °C for 5 min, rinsed in isopropanol and deionized water. Similarly, glass substrates were cleaned using Decon90 glass cleaner, and then rinsed in deionized water. Both the Si and glass substrates were afterwards dried in N_2_ gas atmosphere. Initially, the chamber was evacuated to 6.5 × 10^−6^ Torr, after which the bottom ITO layer was deposited using a RF power of 120 W, with 5.2 mTorr working pressure and 50 sccm Ar gas flow. Before deposition of the ITO top layer (same parameters as for the bottom ITO layer), Al and Ag films were deposited subsequently using DC sputtering with the following sputtering parameters: DC power of 100 W, 5.2 mTorr working pressure and 100 sccm Ar gas flow for the Al film; DC power of 100 W, 4.9 mTorr working pressure and 100 sccm Ar gas flow for the Ag film. The deposition process for all the layers was carried out at room temperature with a target-to-substrate distance of 7 cm.

### Thickness measurements

An optical reﬂectometer Filmetrics F20 was used to measure the film thickness. Each of the deposited thin films of ITO, Al and Ag were measured separately. The measured thicknesses are 37 nm for top and bottom ITO films, 6 nm and 9 nm for Al and Ag films, respectively, and 90 nm for the pure ITO film. For each film, a separate structure on Si was set in the FILMeasure software and all measurements took place in air. A baseline measurement was conducted before each thickness measurement and at least 97.4% of goodness fit was achieved.

### Furnace annealing treatment

A Carbolite electric furnace was used for the annealing of the prepared thin films at 400 °C without any added gases. Prior to annealing, the furnace was at room temperature. A temperature ramp of 10 °C/min was set to reach 400 °C. After stabilization for 5 min, the furnace was let to cool to room temperature.

### Structural analyses of the thin films

The phase composition of the as-deposited and annealed IAAI films was determined using X-ray diffraction. A PANalytic XPERT-PRO MPD X-ray diffractometer model was used with Cu Kα_1_ (λ = 1.540598 Å) radiation, 40 kV working voltage and 30 mA filament current, in a range of 2θ = 15–90°. The Scherrer equation was used to calculate the crystallite (grain) size.

### Elemental composition analyses

The elemental composition of as-deposited and annealed IAAI multilayer films was investigated using energy-dispersive X-ray spectroscopy (EDXS). A FESEM JEOL JSM-7600F, Japan, equipped with an energy-dispersive X-ray spectrometer EDS, OXFORD X-MAX, Energy 200 premium was used.

### Morphological analyses by atomic force microscopic

An AFM Standard Operation AFM5010 Hitachi model in tapping mode was used to examine the surface morphology of the films. Root mean square *R*_rms_ and average *R*_a_ roughness plus morphological grain size analyses were carried out using the Nanoscope Analysis software. All films were scanned over an area of 1 μm × 1 μm.

### Field-emission scanning electron microscopic analyses

The surface morphology of the prepared thin films was further studied using field-emission scanning electron microscopy (FESEM). A FESEM JEOL JSM-7600F (Japan) equipped with an energy-dispersive X-ray spectrometer EDS, OXFORD X-MAX, Energy 200 premium was used. The morphological analyses of the IAAI and ITO films were performed using 5.0 kV voltage and 100000× magnification.

### Optical transmittance analyses

Optical transmittance analyses were carried out using a Shimadzu UV-3101 PC UV–vis double-beam spectrophotometer in the wavelength range of 300–700 nm. IAAI films deposited on glass substrates were used and prior to optical measurements, background (baseline) measurements were performed using two non-doped soda-lime glass samples placed at the two adjacent sample holders.

### Four-point probe measurements

Electrical analyses that involved the measurements of electrical resistivity and sheet resistance of the prepared thin films were performed using a four-point probe system (Pro 4 Lucab Lab). The electrical resistance of the samples was determined at room temperature with a four-point probe taking into account the measured film thickness and a supply current of 4 mA. The results obtained are the average of repeated measurements.

### Hall effect measurements analyses

Further studies on electrical properties in terms of carrier mobility and carrier concentration were carried out using a Hall effect measurement system. During this experiment, the maximum voltage and current were set at 20 V and 20 mA, respectively, using the van der Pauw method by employing a four-point probe situated around the sample perimeter. All samples underwent ohmic contact measurements at room temperature before Hall mobility and carrier concentration measurements.
